# Femoral head avascular necrosis in COVID-19 survivors: a systematic review

**DOI:** 10.1007/s00296-023-05373-8

**Published:** 2023-06-20

**Authors:** Ahmed Abdelazim A. Hassan, Ahmed A. Khalifa

**Affiliations:** 1 Orthopaedic and Traumatology Department, Assiut University Hospital, Assiut, Egypt; 2 Orthopedic Department, Qena Faculty of Medicine, South Valley University, Kilo 6 Qena-Safaga Highway, Qena, Egypt

**Keywords:** Avascular necrosis, Osteonecrosis, Femoral head, Hip joint, COVID-19, Coronavirus, corticosteroids

## Abstract

The current systematic review aimed to document published cases of femoral head avascular necrosis (FHAVN) post-COVID-19, to report the COVID-19 disease characteristics and management patients received, and to evaluate how the FHAVN were diagnosed and treated among various reports. A systematic literature review was performed per the Preferred Reporting Items for Systematic Reviews and Meta-Analyses (PRISMA) guidelines through a comprehensive English literature search on January 2023 through four databases (Embase, PubMed, Cochrane Library, and Scopus), including studies reporting on FHAVN post-COVID-19. Fourteen articles were included, ten (71.4%) were case reports, and four (28.6%) case series reported on 104 patients having a mean age of 42.2 ± 11.7 (14:74) years, in which 182 hip joints were affected. In 13 reports, corticosteroids were used during the COVID-19 management plan for a mean of 24.8 ± 11 (7:42) days, with a mean prednisolone equivalent dose of 1238.5 ± 492.8 (100:3520) mg. A mean of 142.1 ± 107.6 (7:459) days passed between COVID-19 diagnosis and FHAVN detection, and most of the hips were stage II (70.1%), and concomitant septic arthritis was present in eight (4.4%) hips. Most hips (147, 80.8%) were treated non-surgically, of which 143 (78.6%) hips received medical treatment, while 35 (19.2%) hips were surgically managed, 16 (8.8%) core decompression, 13 (7.1%) primary THA, five (2.7%) staged THA and three (1.6%) had first stage THA (debridement and application of antibiotic-loaded cement spacer). The outcomes were acceptable as regards hip function and pain relief. Femoral head avascular necrosis post-COVID-19 infection is a real concern, primarily attributed to corticosteroid usage, besides other factors. Early suspicion and detection are mandatory, as conservative management lines are effective during early stages with acceptable outcomes. However, surgical intervention was required for progressive collapse or patients presented in the late stage.

**Supplementary Information:**

The online version contains supplementary material available at 10.1007/s00296-023-05373-8.

## Introduction

In December 2019, a COVID-19 infection caused by a novel coronavirus was reported from Wuhan City, China. Soon, this disease became a worldwide concern, and after affecting various countries, the WHO declared it a pandemic [1]. At that time, most of the treating physicians and the published reports were concerned by its respiratory system symptoms, especially acute respiratory distress syndrome [2].

Later on, it was evident that COVID-19 affected the whole-body systems leading to and not limited to Guillain-Barré syndrome, lung fibrosis, pulmonary thromboembolism, cardiomyopathy, sensory dysfunction, and stroke [3]. Furthermore, the musculoskeletal system was no exception where patients presented with symptoms such as myalgia and arthralgia up to rhabdomyolysis and joint osteonecrosis, which could be attributed to the systematic inflammatory response induced by COVID-19 [4].

The term “Long COVID” was then introduced to describe the long-term effects of the disease (including persistent arthralgia), which could continue for several weeks or even months after recovering from the COVID-19 acute stage [5]; furthermore, the National Institute for Health and Care Excellence defined it as symptoms lasting more than 12 weeks [6].

Based on the experience after the SARS Epidemic in 2003 where an increase in femoral head avascular necrosis (FHAVN) cases was reported reaching between 23% to 28.8% in the affected patients, which was mainly attributed to massive corticosteroids usage to treat respiratory symptoms, and lowering the inflammatory response [7–10], some authors raised concerns regarding the occurrence of the same scenario with the new COVID-19 pandemic [7, 10–12].

Although avascular necrosis (AVN) or osteonecrosis was reported in various joints, including the knees, shoulders, and spine, the hip joint by far is the most affected [4, 13–16]. However, the literature published on FHAVN is mainly composed of discrete case reports or series, and most of them are limited by the small number of included patients, so collecting and combining the evidence mentioned in these reports could pave the way for better understanding of the causes, diagnosis, and management options for FHAVN. So, the current systematic review aimed to document published cases of FHAVN post-COVID-19, to report the COVID-19 disease characteristics and management patients received, and to evaluate how the FHAVN was diagnosed and treated among various reports.

## Methods

 A systematic literature review was performed per the Preferred Reporting Items for Systematic Reviews and Meta-Analyses (PRISMA) guidelines (supplementary file 1) [17]. It was registered in PROSPERO (registration ID: CRD42023390075).

### Eligibility criteria

The inclusion criteria were English language studies (randomized controlled trials (RCTs), cohort studies, case series, and case reports) reporting on FHAVN post-COVID-19. Studies not published in English, performed on animal models, and other publication types (reviews, editorials, abstracts, and commentaries) were excluded.

We created a search strategy based on a predefined population, intervention/exposure, comparison, and outcome (PI/ECO) model. The population of interest was patients with confirmed FHAVN using plain radiographs and magnetic resonance imaging (MRI) studies. The exposure was COVID-19 (whether the patient was admitted to a hospital or treated at home) and the type of medications the patients received as part of the COVID-19 management protocol. The intervention was how FHAVN was managed (either surgical or non-surgical). No comparison was required. The main outcome parameters were how FHAVN was managed and the final outcomes.

### Information sources and search strategy

A comprehensive English literature search was performed by both authors on January 2023 through four databases (Embase, PubMed, Cochrane Library, and Scopus), using various combinations of the following terms (avascular necrosis of femur head, osteonecrosis of the femoral head, COVID-19, COVID, and coronavirus).

### Selection process

**Fig. 1:**
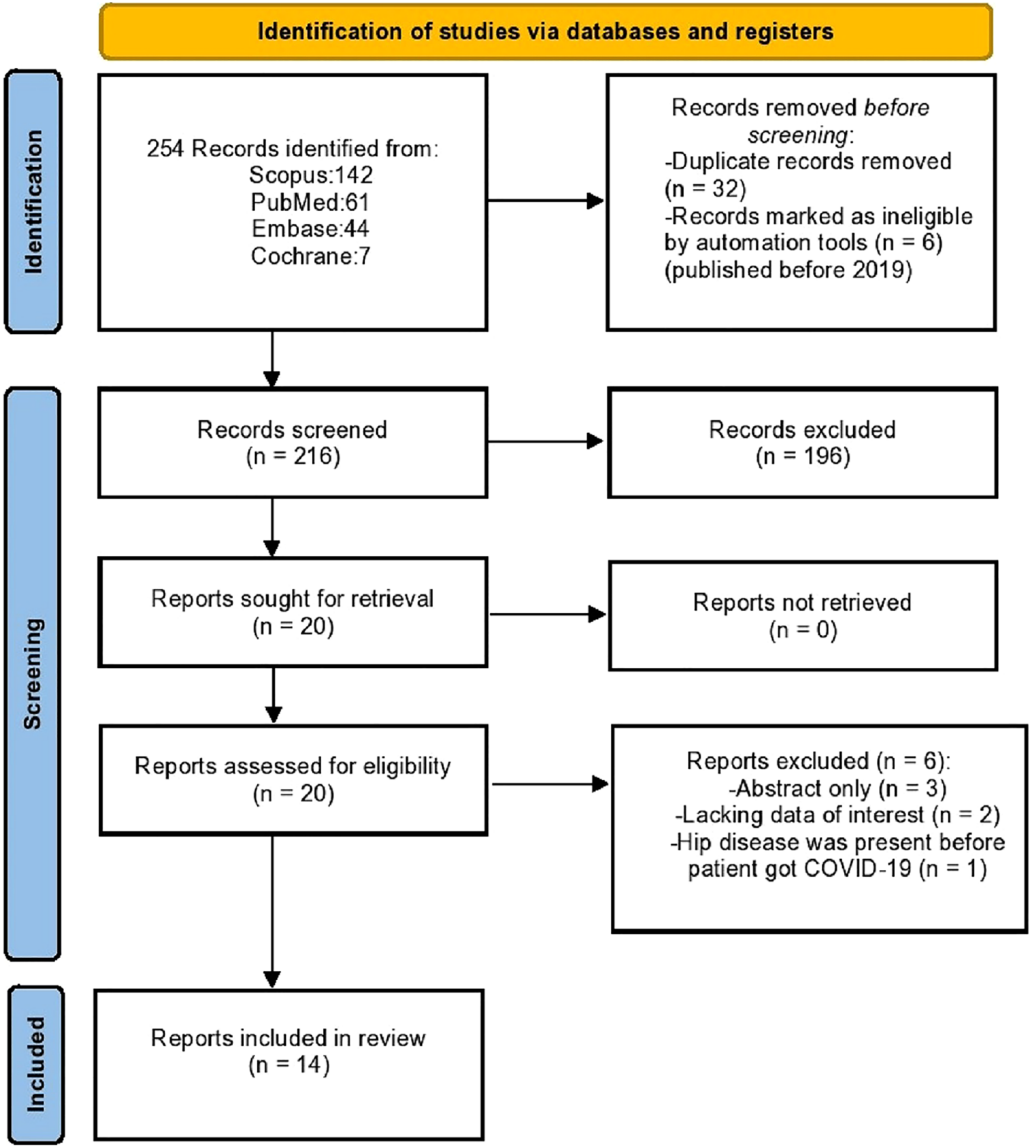
Flow diagram showing the study search and selection method

All the results were downloaded to EndNote 20 program; first, duplicates were removed, then the authors individually evaluated the titles and abstracts of the remaining articles for eligibility. If the title and abstract do not contain enough data, the full text of the article was evaluated. Then the authors discussed the final results, resolved controversies, and decided on the eligible articles. This resulted in the final 14 articles being included in the review synthesis (Fig. 1) [15, 18–30].

The articles were divided equally between the authors; each extracted the data from the articles assigned to him/her; after finishing data extraction, the authors exchanged their data, and each reviewed the data extracted by his/her colleague. If a controversy or missing data were present, a discussion between the authors to resolve any disagreement.

### Data collection and critical appraisal

The following data were extracted: 1- articles characteristics (author (s), Year of publication, country of origin, type of the study). 2-patients characteristics (age, sex, laterality of the disease, comorbidities). 3-COVID-19 infection (diagnosis confirmation- admission to hospital or ICU- the type of management given), if corticosteroids were given, the dose, duration, and route of administration were documented. 4-FHAVN (type of management if surgical or non-surgical; (if surgical, the type of intervention performed will be documented), outcomes (radiographic or functional), and follow up period.

## Results

### Studies and patient characteristics (Table 1)

Of the 14 articles included, ten (71.4%) were case reports, and four (28.6%) case series reported on 104 patients having a mean age of 42.2 ± 11.7 (14:74) years, in which 182 hip joints were affected. The details of publication year, country of origin, patients' gender, and comorbidities are mentioned in Table 1.

**Table 1:** Basic studies and patients characteristics

No	Author	Country	Year of publication	Type of study	No. of patients (no. of hips)	Age (years)*	Gender		Comorbidity	Follow up**
							M	F		
1	Agarwala et al. [19]	India	2021	Case report	3 (5 hips)	37.3 ± 1.3 (36–39)	3	0	NR	70.7 days (30–84)
2	Sulewski et al. [15]	Poland & Switzerland	2021	Case series	3 (3 hip)‡	66.7 ± 3.4 (62–70)	1	2	1 DM	7.3 months (5–10)
3	Yilmam et al. [29]	Turkey	2021	Case report	1 (2 hips)	44	0	1	Congenital Torticollis	NR
4	Agarwala et al. [18]	India	2022	Case series	48 patients (88 hips)	NR	NR	NR	NR	10 months (6–13)
5	Annam et al. [20]	India	2022	Case report	2 (4 hips)	48 ± 21 (27–96)	2	0	NR	NR
6	Ardakani et al. [21]	Iran	2022	Case report	5 (8 hips)	38.4 ± 14.5 (14–54)	2	3	1 ALL, 1 Breast cancer	NR
7	Dhanasekararaja et al. [22]	India	2022	Case series	22 (39 hips)	38.8 (20–74)	20	2	2 DM, 4 HTN, 1 hypothyroidism	NR
8	Etta et al. [30]	India	2022	Case report	1 (2 hips)	38	1	0	Renal transplantation on immunosuppression	NR
9	Jain and Sawant [23]	India	2022	Case report	1 (2 hips)§	42	1	0	None	10 months
10	Kamani et al. [24]	India	2022	Case report	1 (2 hips)	40	1	0	NR	NR
11	Kandari et al. [25]	India	2022	Case series	11 (16 hips)	45.8 ± 8.3 (27–56)	9	2	1 HTN	7 months
12	Kinjima et al. [26]	USA	2022	Case report	1 (2 hips)	60	1	0	Alcoholic, HTN, hyperlipidemia, coronary artery disease	NR
13	Maharjan et al. [27]	Nepal	2022	Case report	1 (2 hips)	22	0	1	NR	2 months
14	Mahran et al. [28]	Egypt	2022	Case report	4 (7 hips)	27.8 ± 6.4 (19–36)	2	2	NR	NR

^‡^The authors included 10 patients in their report (3 of which has only hip joint affection, while the other 7, had other joints affection including four knees, a shoulder, a sacrum and a spine)

^§^The authors reported that the Patient later on developed left knee affection, and the MRI showed bony infarcts of the distal end of the femur and proximal tibia

*Mean ± SD (range), **Mean (range)

*No*.: number, *M*: males, *F* females, *NR* not reported, *DM* diabetes mellitus, *ALL* acute lymphocytic leukemia, *HTN* hypertension

### COVID-19 infection and its management characteristics (Table 2)

Reporting the details on the severity of the disease and the need for ICU admission varied among the included reports. The details of the medical management were mentioned in seven reports [19–21, 23, 25, 29, 30], which included steroids, antivirals, antibiotics, biologics, and anticoagulants. In all reports, corticosteroids were used in the management plan except for one [15], where no steroids were used for any of the patients. The duration of steroid administration had a mean of 24.8 ± 11 (7:42) days, as mentioned in nine reports [19–23, 25, 27–29]. However, the dose of steroid given was mentioned in only six reports [18–22, 25], with a mean prednisolone equivalent of 1238.5 ± 492.8 (100:3520) mg.

**Table 2:** COVID-19 and its management details

No	AUTHOR	ICU Admission	Disease severity	Management	Patients received steroids	Dose of steroid (of prednisolone equivalents)*	Mean Duration of Steroid Administration (days)**	Route of steroid administration
1	Agarwala et al. [19]	Yes (1 patient)	NR	Steroids in all patients + ( IV remedesivir + IV tocilizumab in one Patient, and oral favipiravir in another)	Yes	758 mg (400–1250)	22.5 ( reported for two patients out of the three)	( 2 patients IV then oral, 1 patient oral from the start)
2	Sulewski et al. [15]	NR	1 severe, 1 moderate, 1 mild	-Details were not reported -None of the three patients received steroids	No	NA	NA	NA
3	Yilmam et al. [29]	Yes	NR	Steroids, anti-virals, anti-biotics, and anticoagulants	Yes	NR	45	Oral then IV
4	Agarwala et al. [18]	NR	NR	Details were not reported	Yes	841.3 mg (100–3520)	NR	NR
5	Annam et al. [20]	NR	NR	Steroids, anti-virals, and anti-biotics	Yes	1470 ± 305 mg (430 – 1040)	19 (17–21)	Oral & IV
6	Ardakani et al. [21]	NR	NR	Steroids, anti-viral, biologics, and antibiotics	Yes	1695.2 mg (1375–2010)	16 (10–32)	Oral & IV
7	Dhanasekararaja et al. [22]	NR	NR	Details were not reported	Yes	811 mg (200–2100)	19.6 (7–28)	NR
8	Etta et al. [30]	NR	NR	Steroids, anti-virals, and anticoagulants	Yes	NR	NR	NR
9	Jain and Sawant [23]	NR	NR	Steroids, anti-viral, biologics, anti-biotics, and anticoagulants	Yes	NR	22	IV then oral
10	Kamani et al. [24]	yes	NR	Details were not reported	Yes	NR	NR	IV
11	Kandari et al. [25]	Yes (1 patient)	Moderate and severe	steroids and anti-virals	Yes	1855.6 mg (1600–2400)	22	NR
12	Kinjima et al. [26]	Yes	NR	Details were not reported	Yes	NR	NR	NR
13	Maharjan et al. [27]	NR	NR	Details were not reported	Yes	NR	42	Oral and IV
14	Mahran et al. [28]	Yes (1 patient)	1 severe, 3 moderates	Details were not reported	Yes	NR	14.7 (10–14)	NR

*Mean ± SD (range), **Mean (range)

*NR* not reported, *NA* not applicable, *IV* intravenous, *mg* Milligrams

### Hip joint affection and management characteristics (Table 3)

The laterality of the affected side was reported in all articles except for one [18], 37 bilateral, and 16 unilateral hip affection. The time interval between being diagnosed with COVID-19 infection and FHAVN detection was reported in all studies except for one [24], having a mean of 142.1 ± 107.6 (7:459) days. FHAVN staging was reported in 174 hips from 12 reports [15, 18–28], which was divided into 16 (9.2%) stage I, 122 (70.1%) stage II, 24 (13.8%) stage III 12 (6.9%) stage IV. Noteworthy, in one report, the authors excluded hips classified as stage IV [18]. Furthermore, in eight (4.4%) hips from two reports [21, 23], there was concomitant septic arthritis with AVN caused by various organisms (2 non-typhoid salmonellae, 2 Serratia marcescens, two coagulase-positive staphylococci, and two multidrug-resistant Escherichia coli).

Hip AVN management strategy varied among reports, including conservative (non-surgical) and surgical options. Most hips (147, 80.8%) were treated non-surgically (none of the hips was stage IV), where 143 (78.6%) hips had various combinations of medical treatment [15, 18, 19, 22, 25], two (1.1%) had hyperbaric oxygen therapy accompanied with physiotherapy [29], and two (1.1%) had hip aspiration and irrigation [21].

Thirty-five (19.2%) hips were surgically managed, 16 (8.8%) were treated by core decompression [20, 24, 25, 27, 28, 30]; a concomitant bone graft or bone marrow aspirate was injected in four [27, 30], and a concomitant medical treatment was accompanied in two [27]. First stage THA (debridement and application of antibiotic-loaded cement spacer) was performed in three (1.6%) hips [21]. Primary THA was performed in 13 (7.1%) hips [15, 20, 22, 25, 26], and staged THA was performed in five (2.7%) hips [21, 23]. Four hips were later converted to THA after initial core decompression in one [28], and initial medical treatment in three [22].

The outcomes were reported from eight reports [15, 18, 19, 21–25, 27]; although the measurements used were inconsistent among reports, most of the reports indicated improved functional outcomes and pain relief; details are shown in (Table 3).

**Table 3:** Hip joint affection and management details

No	Author	Side	The time interval between COVID-19 diagnosis and hip AVN detection*	AVNFH stages (Classification)	Management	Outcomes
1	Agarwala et al. [19]	2 Bilateral, 1 left side only	58 ± 1.9 days (45–67)	Stage II in all hips (Ficat-Arlet)	Weekly oral alendronate (70 mg) and annual IV zoledronic acid (5 mg)	Mean VAS improved from 8 (6–9) to 2.7 (1–4) (no patient required surgery)
2	Sulewski et al. [15]	Unilateral	13.3 ± 0.5 days (7–22)	2 Stage IV, 1 stage II (Steinberg scale)	-1 Medical (NSAIDs, intra-articular steroid injections, joint aspiration) -2 THA	VAS improved from (7–8) to (0–2)
3	Yilmam et al. [29]	Bilateral	95 days	NR	Hyperbaric oxygen, and physiotherapy^‡^	NR
4	Agarwala et al. [18]	NR	179 days (59–459)	13 Stage I, 66 stage II, 9 stage III (Ficat-Arlet)	Intravenous zoledronic acid (ZA) injection (5 mg) at the initiation of therapy + oral alendronate (35 mg) twice a week along + calcium (1,000 mg) + vitamin D3 (800 IU) daily, and Partial weight-bearing for the first 3 months	-At six months, VAS improved from 7.03 at presentation to 3.13, and HHS improved from 59.14 at presentation to 71.01 -95.5% of the hips had good clinical outcomes and did not require any surgical intervention
5	Annam et al. [20]	Bilateral	6.5 months (4–9)	1 stage I, 2 stage II, 1 stage IV (Ficat-Arlet)	2 Core decompression & 2 THA	NR
6	Ardakani et al. [21]	3 Bilateral, 1 right side, 1 left side	41.6 ± 1.4 days (29–56)	Reported 4 hips, 2 Stage IV, 2 stage II (Ficat-Arlet)^§^	-Hip aspiration was performed for all hips to exclude infection -Aspiration and irrigation for 2 hips -1st stage THA only in three hips -staged THA in three hips	Mean VAS improved from 9.4 to 2.8
7	Dhanasekararaja et al. [22]	17 Bilateral, 2 right side, 3 left side	7.5 months (3–11)	32 Stage II, 7 stage III (Ficat-Arlet)	-All the patients underwent initial conservative therapy with bisphosphonates, calcium and non-steroidal anti-inflammatory agents -3 hips diagnosed with Rapidly destructive coxarthrosis underwent THA	Improved HHS ( from 63.6 ± 23.2 at presentation to 82.6 ± 9.6 after treatment)
8	Etta et al. [30]	Bilateral	63 days	NR	Core decompression with bone grafting and physiotherapy	NR
9	Jain and Sawant [23]	Bilateral	25 days	Stage IV (Ficat-Arlet)^**^	Staged THA	Full painless range of motion of both hips with no clinical signs of active infection
10	Kamani et al. [24]	Bilateral	NR	STAGE IV and II (Ficat-Arlet)	Core decompression and physiotherapy	The numerical Pain Rating Scale (NPRS) improved from 8/10 to 2/10
11	Kandari et al. [25]	5 Bilateral, 6 unilateral	7 months	1 Stage I, 8 stage II, 5 stage III, 2 stage IV (Ficat-Arlet)	2 THA, 1 core decompression, 13 conservative (bisphosphonates and protected weight bearing)	-Mean VAS improved from 7.19 ± 0.91 to 2.69 ± 1.16 -Mean HHS improved from 59.25 ± 6.92 to 77.75 ± 5.91
12	Kinjima et al. [26]	Bilateral	10 months	Stage IV (Ficat-Arlet)	THA	NR
13	Maharjan et al. [27]	Bilateral	7 months	Stage I and stage III (Ficat-Arlet)	Core decompression, BMAC injection, physiotherapy, and bisphosphonates (alendronate 70 mg OD weekly for 2 months)	Clinical improvement was reported (no scores reported); patient was mobilizing without crutches
14	Mahran et al. [28]	3 Bilateral, 1 left side	10.3 months (7–12)	5 Stage II, 2 stage III (Steinberg scale)	Core decompression (with only one later conversion to THA)	NR

^‡^The authors mentioned that this line of management was planned for the Patient; however, they did not report if the Patient had it or not

^§^Concomitant infection was reported in 6 hips (2 non-typhoid salmonellae, 2 Serratia marcescens, and 2 coagulase-positive staphylococci)

**Concomitant infection in both hips (multidrug-resistant Escherichia coli)

*Mean ± SD (range)

-Staged THA: this means that the Patient had a cement spacer loaded with antibiotics as a first stage, then the second stage was definitive THA

*AVNFH* avascular necrosis of the femoral head, *NR* not reported, THA total hip arthroplasty, *VAS* visual analog scale, HHS Harris hip score

## Discussion

The current review confirmed the concerns some authors raised about FHAVN development in patients recovering from COVID-19 infection; most of the reports related this to corticosteroid administration as part of the COVID-19 management plan; however, other factors were suggested. In all reports, MRI scans were mandatory to confirm the diagnosis. In most hips, conservative management was adequate with acceptable outcomes; however, surgical intervention was required for progressive collapse or patients presented in the late stage.

Femoral head avascular necrosis (FHAVN) (which is synonymously called osteonecrosis) is a disease where the femoral head vascularity is deficient, leading to the death of the osteocytes and bone marrow [31, 32], which could occur primarily where no definitive cause is detected or secondary, which mainly occur after excessive steroids usage or alcohol consumption [31, 33]. Usually, this condition affects young adults, and bilateral hip affection could occur in up to 75% of cases [31, 34]. Although it could be evident in plain hip radiographs, MRI is the imaging modality of choice for early diagnosis and proper staging for management planning [31, 32].

Since the emergence of COVID-19 infection, scientists and physicians rushed to find proper management to slow down the morbidity and mortality rates, of the drugs used were antivirals, angiotensin receptor blockers, chloroquine phosphate, and corticosteroids [35, 36]; the latter showed efficacy in managing acute respiratory distress syndrome symptoms and preventing acute lung injury; furthermore, administration of 6 mg dexamethasone per day for ten days showed to reduce the overall 28-day mortality rate according to the study by the RECOVERY group [37]. However, the benefits of corticosteroid use came at the expense of resulting in certain steroid-related complications such as bone marrow osteonecrosis, secondary diabetes mellitus, and secondary infections [38].

## A-Theories behind FHAVN development in patients post-COVID-19 infection

### 1-Corticosteroid induced

By far, corticosteroid-induced FHAVN is the most commonly adopted explanation; in the current review, 13 out of the 14 reports [18–30], corticosteroids were part of the COVID-19 management protocol, and the authors in these reports alluded to the possible contribution of corticosteroids in FHAVN development, however, in only one report by Kandari et al. [25], the authors confirmed a direct link between corticosteroids dose and administration duration and the FHAVN severity and staging.

There have been many studies relating FHAVN development with massive corticosteroid usage [12, 19, 39], and various suggested mechanisms for this correlation were proposed, such as the possibility of fat emboli formation, hyperlipidemia, endothelial dysfunction, hypoxia, hypercoagulable status, and increased intraosseous pressure secondary to fat cell hypertrophy, all of these factors contributed to bone marrow ischemia and eventual bone necrosis [40, 41], however, the actual pathogenesis still not well-established [42].

Although corticosteroid was the most blamed single factor for FHAVN development, there is a lack of consensus regarding the least causative dose, route, and duration of administration needed for AVN to develop. In the current review, the mean prednisolone equivalent was 1238.5 ± 492.8 mg, which was consistent with what was reported in the literature that a minimum dose ranging from 700 to 2000 mg prednisone, or its equivalent, was required for FHAVN development [39, 41]. After SARS, patients younger than 50 years who received corticosteroids of more than 3000 mg for a mean duration of 25 days were considered candidates for FHAVN development [9]. In a 17-year follow up study by Sing et al., the authors reported a clear association between high-dose steroids (even if administered for a short course) and FHAVN development [43].

Even more, FHAVN after exposure to low doses of corticosteroids had been reported in the literature, which was as minimum as 40 mg [10]; in the current review, the minimum corticosteroid dose was 100 mg, as reported in Agarwala et al. series [18].

In the current review, the mean duration of corticosteroid administration was 24.8 ± 11 days, consistent with 25 days mean duration reported in patients recovering from SARS [9]. Chan et al. reported a shorter duration of about 18 days of methylprednisolone (> 2000 mg) administration, resulting in FHAVN prevalence of up to 10% [44]. In a study that included 1137 patients with SARS, Zhao et al. confirmed that osteonecrosis was related to the corticosteroid administration duration, as the osteonecrosis rate ratio was 1.29 for every ten days of treatment; they recommended that modifying the corticosteroid administration duration will lower the risk of osteonecrosis [45].

Furthermore, the time interval between corticosteroid administration to FHAVN development ranged between 6 to 12 months [46, 47]. However, some authors reported "very early" steroid-induced FHAVN, which could develop after one to three months [48, 49]. In the current review, we found that a mean of 142.1 ± 107.6 days (about 4.7 months) was needed for FHAVN to be detected after COVID-19 diagnosis, which is considered a relatively shorter interval than what was reported in the literature.

### II-Other possible causes explaining FHAVN development

In the current review, no corticosteroid was administered by any of the patients included in the series by Sulewski et al. [15]; however, they reported osteonecrosis in ten patients, including three FHAVN. This shed light on the possibility of other contributing factors for FHAVN development.

Some of the possible causes proposed by some authors are as follows. First, during the systemic inflammation response to COVID-19, Cytokines such as IL-17 and TNF-alpha are produced; this response reduces osteoblast proliferation and differentiation [50]. Second, Viral infection leading to angiotensin-converting enzyme 2 (ACE2) deficiency could induce bone matrix degradation [51, 52]. Third, a hypercoagulable state in COVID-19 patients caused by the systematic inflammatory response accompanied by direct endothelial injury could increase bone necrosis possibility [53, 54]. This was confirmed in some cadaveric studies, which showed coagulopathy and intravascular thrombosis in patients post-COVID-19 [55]. Last but not least, the antiviral therapy as reported by Ardoy and Aguilera, where they had a patient presented with knee medial condyle osteonecrosis after having COVID-19 infection [13], the authors based their suggestion on previous experience with SARS 2003 [9]; however, this suggestion should be considered cautiously, as the antivirals used in COVID-19 are different from those with SARS [56].

## B- differential diagnosis of hip pain post-COVID-19 infections

Patients could present with arthralgia as part of the COVID-19 musculoskeletal consequences [4]; other causes of hip joint pain rather than FHAVN should be identified for proper management.

One serious hip condition which could present in patients recovering from COVID-19 is septic arthritis, which was found concomitant with FHAVN in the current review in eight hips, as documented in two reports [21, 23]. This was suspected by elevated inflammatory markers accompanying edema and collection in the soft tissues around the hip, as shown in the MRI images. For this concern, Ardakani et al. reported performing aspiration for all of their patients [21], and Dhanasekararaja et al. reported performing aspiration for selected patients, especially when patients present with acute and aggressive clinical manifestations, including severe pain and elevated serological markers, and if the MRI showed a rapid collapse of the femoral head, soft tissue edema and collection [22].

A second possibility is the presence of reactive arthritis, which could develop following viral infection, and usually develops a few weeks after the infection and lacks the MRI finding of the FHAVN [57]. The last but weak possibility is the development of viral arthritis, which was reported with other viral infections such as HCV and HIV; however, this type is challenging to diagnose and presents as poly-articular affection [58].

## C-Management options for FHAVN

### I-Early detection and follow up of patients at risk

The aim is to diagnose the AVN as early as possible before it progresses to advanced stages, as up to 97% of the hips could avoid having surgical intervention if diagnosed and managed during stages I or II [59]. During the early stages, the management plan aims mainly at alleviating pain, improving function, prevent further progression and femoral head collapse; this could be achieved by various options such as medical treatment, physiotherapy, and hyperbaric oxygen therapy [60, 61]. However, if the disease is progressive and the conservative lines have no role, surgical intervention in the form of core decompression with or without bone grafting or total hip arthroplasty (THA) is necessary [31].

Zhang and Zhang suggested applying a risk stratification strategy for patients who had COVID-19 infection for the early detection of FHAVN, which was based on the recommendations by the ARCO committee China branch; they suggested dividing the patients into three main categories: 1-low-risk, if the patients did not receive corticosteroids, 2-moderate-risk, if less than 2000 mg corticosteroids were administered and for less than a week, and 3-high-risk, if more than 2000 mg corticosteroids were received for more than one week or if the patient received IV pulse more than 80 mg per day for at least three days [10, 62, 63].

They recommended tailoring the follow up plan according to the risk category, where patients recovering from COVID-19 should be followed for at least 24 months, with MRI being the diagnostic modality of choice for screening and early detection; furthermore, the high-risk group should attend regular clinic follow up at two to six months intervals after being exposed to corticosteroids, while the frequency is less for the low and moderate risk groups [62, 63]. From the articles included in the current review, some authors recommended performing early MRI for patients recovering from COVID-19 infection if they started feeling hip joint discomfort [18, 22, 28].

### II-Definitive management for established cases

#### 1) Non-surgical management

Various modalities were suggested for non-surgical intervention management options for early detected FHAVN, including protected weight bearing, physical therapy, vasodilators, oral anticoagulants, traditional Chinese therapy, and bisphosphonates [12, 62, 63]. Furthermore, reports on FHAVN post-SARS showed that the osteonecrotic lesions tend to reduce in size and stabilize over time, unlike AVN developed after steroid usage in other conditions such as autoimmune diseases [45, 64].

Most of the hips included in the current review (80.8%) were managed non-surgically, where 143 (78.6%) hips had various combinations of medical treatment, as shown in (Table 3), which was successful in controlling the progression of FHAVN, with acceptable outcomes. Noteworthy, in the largest included series by Agarwala et al. [18], the authors excluded patients diagnosed with FHAVN stage IV from being candidates for medical management; furthermore, the authors reported that 95.5% of their patients reported good outcomes and disease progression necessitating surgical intervention in the form of THA was reported in only four hips.

#### 2) Surgical intervention

In cases where the FHAVN is advanced (stage III or IV) or when the disease progress rapidly after initial conservative management, various surgical options could be performed, including core decompression, bone grafting, and arthroplasty [31, 34].

In a study by Guo et al. retrospectively evaluated 539 patients treated for SARS, they reported that steroid-related FHAVN reached up to 24.1%, and 7.8% of the hips required surgical intervention [65]. On the contrary, in the current review, a higher percentage needed surgical intervention, where 35 (19.5%) hips were treated by different modalities, which mostly changed according to the presence of concomitant hip joint pathology. Core decompression was the most common surgical intervention, performed in 16 (8.8%) hips, followed by primary THA in 13 (7.1%) hips; however, in one hip which had initial core decompression, the disease progressed, and THA was performed. In confirmed or suspected cases of concomitant septic arthritis, a staged procedure was performed, where the first stage consisted of debridement and application of a cement spacer loaded with antibiotics; this was performed in eight (4.4%) hips, five of them, later on, had a second stage conversion to THA.

The current review has some limitations and weaknesses. First, all the included studies are either case reports or case series, and most are formed of small sample sizes; furthermore, this could be a source of bias. Second, the inconsistency and shortage while reporting the exact COVID-19 management plan hindered the evaluation of other possible contributing factors for FHAVN development. Third, the relatively short follow up duration, however, could be attributed to the novelty of the condition. Last, shortage of reporting detailed outcomes after FHAVN management by various options.

Despite the limitations and weaknesses mentioned above, we believe that the main strength of the current review is related to the fact that this was the first systematic review to collect most of the discrete published literature on FHAVN in patients who survived COVID-19, enabling physicians and surgeons dealing with suspected cases to understand the possible risk factors for FHAVN development even in the absence of steroid administration and to clarify that early detection enables the successfulness of conservative management lines.

## Conclusion

Femoral head avascular necrosis in patients surviving COVID-19 is now well documented in the literature, as reported by authors from different countries, and most of them accused corticosteroid usage of causing this problem. However, other possible causes, including hypercoagulable status and the secondary systematic inflammatory response, could be involved, making FHAVN development a multifactorial problem. The disease affects various age groups and could be presented bilaterally. Septic arthritis is a real concern that should be excluded if suspected. In most cases, early detection using an MRI scan and conservative management could preserve the hip joint, prevent further progression and result in acceptable outcomes. However, for patients presenting late, surgical intervention is the management option of choice. A follow up protocol is mandatory for patients surviving COVID-19, especially those who received corticosteroids, and MRI as early as the patients develop hip joint discomfort is advisable to detect early FHAVN development.

## Supplementary Information

Below is the link to the electronic supplementary material.

Supplementary file1 (PDF 69 KB)

## Data Availability

All the data related to the study are mentioned within the manuscript; however, the raw data are available with the corresponding author and will be provided upon a written request.

## References

[1] Cucinotta D, Vanelli M (2020). WHO Declares COVID-19 a Pandemic. Acta Biomed.

[2] Umakanthan S, Sahu P, Ranade AV, Bukelo MM, Rao JS, Abrahao-Machado LF, Dahal S, Kumar H, Kv D (2020). Origin, transmission, diagnosis and management of coronavirus disease 2019 (COVID-19). Postgrad Med J.

[3] Leung TYM, Chan AYL, Chan EW, Chan VKY, Chui CSL, Cowling BJ, Gao L, Ge MQ, Hung IFN, Ip MSM, Ip P, Lau KK, Lau CS, Lau LKW, Leung WK, Li X, Luo H, Man KKC, Ng VWS, Siu CW, Wan EYF, Wing YK, Wong CSM, Wong KHT, Wong ICK (2020). Short- and potential long-term adverse health outcomes of COVID-19: a rapid review. Emerg Microbes Infect.

[4] Disser NP, De Micheli AJ, Schonk MM, Konnaris MA, Piacentini AN, Edon DL, Toresdahl BG, Rodeo SA, Casey EK, Mendias CL (2020). Musculoskeletal Consequences of COVID-19. J Bone Joint Surg Am.

[5] Aiyegbusi OL, Hughes SE, Turner G, Rivera SC, McMullan C, Chandan JS, Haroon S, Price G, Davies EH, Nirantharakumar K, Sapey E, Calvert MJ, Group TLCS (2021). Symptoms, complications and management of long COVID: a review. J R Soc Med.

[6] Mahase E (2020). Covid-19: What do we know about “long covid”?. BMJ.

[7] Zhang S, Wang C, Shi L, Xue Q (2021). Beware of Steroid-induced avascular necrosis of the femoral head in the treatment of COVID-19-experience and lessons from the SARS epidemic. Drug Des Devel Ther.

[8] Auyeung TW, Lee JS, Lai WK, Choi CH, Lee HK, Lee JS, Li PC, Lok KH, Ng YY, Wong WM, Yeung YM (2005). The use of corticosteroid as treatment in SARS was associated with adverse outcomes: a retrospective cohort study. J Infect.

[9] Patel MS, Gutman MJ, Abboud JA (2020). Orthopaedic considerations following COVID-19: lessons from the 2003 SARS Outbreak. JBJS Rev.

[10] Li W, Huang Z, Tan B, Chen G, Li X, Xiong K, Zhu R, Li R, Li S, Ye H, Liang Z, Dong X, Zhou S, Chen S, Xi H, Cheng H, Xu R, Tu S, Chen Z, Qi L, Song J, Xiao R, Liu H, Nan Q, Yu H, Cui H, Shen Y, Wang C, Lin N, Zhang Y, Chen W (2021). General recommendation for assessment and management on the risk of glucocorticoid-induced osteonecrosis in patients with COVID-19. J Orthop Translat.

[11] Snowden GT, Clement ND, Zhang S, Xue Q, Simpson A (2022). Orthopaedic long COVID - the unknown unknowns : are we facing a pandemic of avascular necrosis following COVID-19?. Bone Joint Res.

[12] Shetty GM (2022). Double trouble-COVID-19 and the widespread use of corticosteroids: Are We staring at an osteonecrosis epidemic?. Indian J Orthop.

[13] Angulo-Ardoy M, Urena-Aguilera A (2021). Knee osteonecrosis after COVID-19. Fam Pract.

[14] Ghosh S, Gupta SS, Mehta N, Khodaiji S (2021). COVID-19-Associated bone marrow necrosis-a case report. Indian J Radiolo Imag.

[15] Sulewski A, Sieron D, Szyluk K, Dabrowski M, Kubaszewski L, Lukoszek D, Christe A (2021). Avascular necrosis bone complication after active COVID-19 infection: preliminary results. Medicina (Kaunas).

[16] Baimukhamedov C, Botabekova A, Lessova Z, Abshenov B, Kurmanali N (2023). Osteonecrosis amid the COVID-19 pandemic. Rheumatol Int.

[17] Page MJ, McKenzie JE, Bossuyt PM, Boutron I, Hoffmann TC, Mulrow CD, Shamseer L, Tetzlaff JM, Akl EA, Brennan SE, Chou R, Glanville J, Grimshaw JM, Hrobjartsson A, Lalu MM, Li T, Loder EW, Mayo-Wilson E, McDonald S, McGuinness LA, Stewart LA, Thomas J, Tricco AC, Welch VA, Whiting P, Moher D (2021). The PRISMA 2020 statement: an updated guideline for reporting systematic reviews. Syst Rev.

[18] Agarwala S, Vijayvargiya M, Sawant T, Kulkarni S (2022). Bisphosphonates for Post-COVID osteonecrosis of the femoral head: medical management of a surgical condition. JB JS Open Access.

[19] Agarwala SR, Vijayvargiya M, Pandey P (2021). Avascular necrosis as a part of ‘long COVID-19’. BMJ Case Rep.

[20] Annam P, Manda A, Myneni UK, Sahar AN, Prasad N, Sam KK, Sahu S, Reddy KK (2022). Corticosteroids induced avascular necrosis of hip, a “long COVID-19” complication: case report. Ann Med Surg (Lond).

[21] Ardakani MV, Parviz S, Ghadimi E, Zamani Z, Salehi M, Firoozabadi MA, Mortazavi SMJ (2022). Concomitant septic arthritis of the hip joint and femoral head avascular necrosis in patients with recent COVID-19 infection: a cautionary report. J Orthop Surg Res.

[22] Dhanasekararaja P, Soundarrajan D, Kumar KS, Pushpa BT, Rajkumar N, Rajasekaran S (2022). Aggressive presentation and rapid progression of osteonecrosis of the femoral head after COVID-19. Indian J Orthop.

[23] Jain S, Sawant T (2022). Osteonecrosis with Concomitant bacterial osteomyelitis of both hips and a Knee in a post-COVID-19 patient: a case report. JBJS Case Connect.

[24] Kamani S, Lakhwani MG, Phansopkar P (2022). Undiagnosed bilateral avascular necrosis of the femur in a young male caused by COVID-19 steroid injections. Cureus.

[25] Kandari AKS, Bhamare DS, Salunkhe R, Sukrethan SV, Shevate I, Deshmukh A, Pisal T, Kulkarni K, Janapamala K (2022). Femur head necrosis as a post-acute sequela of Covid-19 (SARS-CoV-2 infection). Genij Ortopedii.

[26] Kingma TJ, Hoch V, Johnson C, Chaudhry B (2022). Avascular necrosis of the Hip: a post COVID-19 sequela. Cureus.

[27] Maharjan G, Yadav S, Yadav MK, Khati N, Bhattarai HB, Joshi J (2022). Steroid-induced avascular necrosis: A case report on a patient treated with steroid therapy for COVID-19. Ann Med Surg (Lond).

[28] Mahran MA, Moustafa MM, Hassan AAA, Bakr H, Abdelaal AM, Khalifa AA (2022) FEMORAL HEAD OSTEONECROSIS POST-COVID-19 INFECTION, A PROPHECY COMING TRUE. A REPORT of SEVEN HIPS. Journal of Musculoskeletal Research (no pagination)

[29] Yilmam I, Kaya BS, Edis EC, Ustabasioglu FE, Copuroglu C (2021). A Case with avascular Bone necrosis developing as a complication of COVID-19 treatment. Respiratory Case Reports.

[30] Etta PK, Madhavi T, Panjwani RS (2022). Coronavirus disease 2019 could be a novel risk factor for avascular necrosis after kidney transplantation. Indian Journal of Transplantation.

[31] Baig SA, Baig MN (2018). Osteonecrosis of the Femoral Head: Etiology. Investigations, and Management Cureus.

[32] Petek D, Hannouche D, Suva D (2019). Osteonecrosis of the femoral head: pathophysiology and current concepts of treatment. EFORT Open Rev.

[33] Shah KN, Racine J, Jones LC, Aaron RK (2015). Pathophysiology and risk factors for osteonecrosis. Curr Rev Musculoskelet Med.

[34] Moya-Angeler J, Gianakos AL, Villa JC, Ni A, Lane JM (2015). Current concepts on osteonecrosis of the femoral head. World J Orthop.

[35] Ip A, Ahn J, Zhou Y, Goy AH, Hansen E, Pecora AL, Sinclaire BA, Bednarz U, Marafelias M, Sawczuk IS, Underwood JP, Walker DM, Prasad R, Sweeney RL, Ponce MG, La Capra S, Cunningham FJ, Calise AG, Pulver BL, Ruocco D, Mojares GE, Eagan MP, Ziontz KL, Mastrokyriakos P, Goldberg SL (2021). Hydroxychloroquine in the treatment of outpatients with mildly symptomatic COVID-19: a multi-center observational study. BMC Infect Dis.

[36] Dhakal N, Poudyal A, Gyanwali P (2021). Pharmacological treatment for the management of COVID 19: A narrative review. JNMA J Nepal Med Assoc.

[37] Horby P, Lim WS, Emberson JR, Mafham M, Bell JL, Linsell L, Staplin N, Brightling C, Ustianowski A, Elmahi E, Prudon B, Green C, Felton T, Chadwick D, Rege K, Fegan C, Chappell LC, Faust SN, Jaki T, Jeffery K, Montgomery A, Rowan K, Juszczak E, Baillie JK, Haynes R, Landray MJ, Group RC (2021). Dexamethasone in Hospitalized Patients with Covid-19. N Engl J Med.

[38] Marte JL, Toney NJ, Cordes L, Schlom J, Donahue RN, Gulley JL (2020). Early changes in immune cell subsets with corticosteroids in patients with solid tumors: implications for COVID-19 management. J Immunotherapy Cancer.

[39] Mont MA, Pivec R, Banerjee S, Issa K, Elmallah RK, Jones LC (2015). High-dose corticosteroid use and risk of hip osteonecrosis: meta-analysis and systematic literature review. The J Arthroplasty.

[40] Xie XH, Wang XL, Yang HL, Zhao DW, Qin L (2015). Steroid-associated osteonecrosis: Epidemiology, pathophysiology, animal model, prevention, and potential treatments (an overview). J Orthop Translat.

[41] Chan KL, Mok CC (2012). Glucocorticoid-induced avascular bone necrosis: diagnosis and management. Open Orthop J.

[42] Wang H, Niu L (2021). Can femoral head necrosis induced by steroid therapy in patients infected with coronaviruses be reversed?. Bone Res.

[43] Sing CW, Tan KCB, Wong ICK, Cheung BMY, Cheung CL (2021). Long-term outcome of short-course high-dose glucocorticoids for severe acute respiratory syndrome (SARS): A 17-Year follow-up in SARS survivors. Clin Infect Dis.

[44] Chan MH, Chan PK, Griffith JF, Chan IH, Lit LC, Wong CK, Antonio GE, Liu EY, Hui DS, Suen MW, Ahuja AT, Sung JJ, Lam CW (2006). Steroid-induced osteonecrosis in severe acute respiratory syndrome: a retrospective analysis of biochemical markers of bone metabolism and corticosteroid therapy. Pathology.

[45] Zhao R, Wang H, Wang X, Feng F (2017). Steroid therapy and the risk of osteonecrosis in SARS patients: a dose-response meta-analysis. Osteoporos Int.

[46] Chang C, Greenspan A, Gershwin ME (2020). The pathogenesis, diagnosis and clinical manifestations of steroid-induced osteonecrosis. J Autoimmun.

[47] Birla V, Vaish A, Vaishya R (2021). Risk factors and pathogenesis of steroid-induced osteonecrosis of femoral head - a scoping review. J Clin Orthop Trauma.

[48] Nagasawa K, Tada Y, Koarada S, Horiuchi T, Tsukamoto H, Murai K, Ueda A, Yoshizawa S, Ohta A (2005). Very early development of steroid-associated osteonecrosis of femoral head in systemic lupus erythematosus: prospective study by MRI. Lupus.

[49] Yildiz N, Ardic F, Deniz S (2008). Very early onset steroid-induced avascular necrosis of the hip and knee in a patient with idiopathic thrombocytopenic purpura. Intern Med.

[50] Huang C, Wang Y, Li X, Ren L, Zhao J, Hu Y, Zhang L, Fan G, Xu J, Gu X, Cheng Z, Yu T, Xia J, Wei Y, Wu W, Xie X, Yin W, Li H, Liu M, Xiao Y, Gao H, Guo L, Xie J, Wang G, Jiang R, Gao Z, Jin Q, Wang J, Cao B (2020). Clinical features of patients infected with 2019 novel coronavirus in Wuhan. China Lancet.

[51] Salvio G, Gianfelice C, Firmani F, Lunetti S, Balercia G, Giacchetti G (2020). Bone metabolism in SARS-CoV-2 disease: possible osteoimmunology and gender implications. Clin Rev Bone Miner Metab.

[52] Tao H, Bai J, Zhang W, Zheng K, Guan P, Ge G, Li M, Geng D (2020). Bone biology and COVID-19 infection: Is ACE2 a potential influence factor?. Med Hypotheses.

[53] Liu J, Li S, Liu J, Liang B, Wang X, Wang H, Li W, Tong Q, Yi J, Zhao L, Xiong L, Guo C, Tian J, Luo J, Yao J, Pang R, Shen H, Peng C, Liu T, Zhang Q, Wu J, Xu L, Lu S, Wang B, Weng Z, Han C, Zhu H, Zhou R, Zhou H, Chen X, Ye P, Zhu B, Wang L, Zhou W, He S, He Y, Jie S, Wei P, Zhang J, Lu Y, Wang W, Zhang L, Li L, Zhou F, Wang J, Dittmer U, Lu M, Hu Y, Yang D, Zheng X (2020). Longitudinal characteristics of lymphocyte responses and cytokine profiles in the peripheral blood of SARS-CoV-2 infected patients. EBioMedicine.

[54] Kichloo A, Dettloff K, Aljadah M, Albosta M, Jamal S, Singh J, Wani F, Kumar A, Vallabhaneni S, Khan MZ (2020). COVID-19 and hypercoagulability: a review. Clin Appl Thromb Hemost.

[55] Fahmy OH, Daas FM, Salunkhe V, Petrey JL, Cosar EF, Ramirez J, Akca O (2021). Is Microthrombosis the main pathology in coronavirus disease 2019 severity?-a systematic review of the postmortem pathologic findings. Crit Care Explor.

[56] Sreekanth Reddy O, Lai WF (2021). Tackling COVID-19 using remdesivir and favipiravir as therapeutic options. ChemBioChem.

[57] Shokraee K, Moradi S, Eftekhari T, Shajari R, Masoumi M (2021). Reactive arthritis in the right hip following COVID-19 infection: a case report. Trop Dis Travel Med Vaccines.

[58] Marks M, Marks JL (2016). Viral arthritis. Clin Med (Lond).

[59] Agarwala S, Banavali SD, Vijayvargiya M (2018). Bisphosphonate combination therapy in the management of postchemotherapy avascular necrosis of the femoral head in adolescents and young adults: a retrospective study from India. J Glob Oncol.

[60] Agarwala S, Shah S, Joshi VR (2009). The use of alendronate in the treatment of avascular necrosis of the femoral head: follow-up to eight years. J Bone Joint Surg Br.

[61] Roth A, Maus U (2022). Drug treatment of osteonecrosis. Orthopadie (Heidelb).

[62] Zhang B, Zhang S (2020). Corticosteroid-Induced Osteonecrosis in COVID-19: A Call For Caution. J Bone Miner Res.

[63] Bone C, Osteonecrosis Professional Committee Shockwave Medical Specialty Committee Of Chinese Research Hospital A (2020). Expert consensus on prevention and treatment strategies for osteonecrosis of femoral head during the prevention and control of novel coronavirus pneumonia (2020). Zhongguo Xiu Fu Chong Jian Wai Ke Za Zhi.

[64] Zhang P, Li J, Liu H, Han N, Ju J, Kou Y, Chen L, Jiang M, Pan F, Zheng Y, Gao Z, Jiang B (2020). Long-term bone and lung consequences associated with hospital-acquired severe acute respiratory syndrome: a 15-year follow-up from a prospective cohort study. Bone Res.

[65] Guo KJ, Zhao FC, Guo Y, Li FL, Zhu L, Zheng W (2014). The influence of age, gender and treatment with steroids on the incidence of osteonecrosis of the femoral head during the management of severe acute respiratory syndrome: a retrospective study. Bone Joint J.

